# Influence of psoriasis on infection risk and survival outcomes in patients with head and neck cancer: a retrospective cohort study

**DOI:** 10.1186/s12885-025-13920-8

**Published:** 2025-03-24

**Authors:** Lin Jian, Mu-Kuan Chen, Chew-Teng Kor, Yen-Tze Liu

**Affiliations:** 1 Department of Dermatology, Changhua Christian Hospital, Changhua, Taiwan; 2 Department of Otorhinolaryngology, Head and Neck Surgery, Changhua Christian Hospital, Changhua, Taiwan; 3 Department of Post-Baccalaureate Medicine, College of Medicine, National Chung Hsing University, Taichung, Taiwan; 4 Big Data Center, Changhua Christian Hospital, No. 135, Nanxiao Street, Changhua, 50006 Taiwan; 5 Graduate Institute of Clinical Medicine, College of Medicine, National Chung Hsing University, Taichung, Taiwan; 6 Department of Family Medicine, Changhua Christian Hospital, Changhua, Taiwan

**Keywords:** Psoriasis, Head and Neck Neoplasms, Infection, Survival, Systemic Therapies

## Abstract

**Background:**

Psoriasis is a chronic inflammatory skin condition mediated by autoimmune processes, which may heighten the susceptibility to infections. However, its impact on infection risk and survival outcomes in patients with head and neck cancer (HNC) remains understudied.

**Methods:**

We conducted a retrospective cohort study using data from a tertiary referral center in Taiwan between January 2010 and August 2021. A total of 4,476 HNC patients were identified, of whom 49 had psoriasis and 4,427 did not. After propensity score matching (PSM), 48 patients with psoriasis and 480 without psoriasis were included in the final analysis. The primary outcome was the one-year post-treatment infection rate, assessed using hazard ratios (HRs) derived from Cox proportional hazards models. Secondary outcomes included overall survival (OS) and disease-free survival (DFS). Subgroup and sensitivity analyses were performed based on psoriasis severity and systemic therapy use.

**Results:**

The one-year infection rate was significantly higher in the psoriasis group compared to the non-psoriasis group (33.3% vs. 20.2%, *P* = 0.035), with a hazard ratio (HR) of 1.84 (95% CI: 1.09–3.11). Psoriasis patients on systemic therapy had an elevated infection risk (HR: 1.99, 95% CI: 1.12–3.53, *P* = 0.0189). Sensitivity analysis confirmed a consistent association between psoriasis and infection risk (HR: 2.04, 95% CI: 1.18–3.51, *P* = 0.0106). Psoriasis did not significantly impact survival outcomes.

**Conclusions:**

Psoriasis is associated with an increased one-year infection risk following HNC treatment, particularly in patients receiving systemic therapy. This finding suggests a need for heightened infection monitoring and preventive care in HNC patients with psoriasis.

## Introduction

Psoriasis is an immune-mediated inflammatory skin disease characterized by erythema and silvery scales [1, 2]. Beyond dermatological manifestations, it is linked to elevated risks of oral cavity, laryngeal, colorectal, pancreatic, nonmelanoma skin cancers, and lymphoma [3, 4]. Proposed mechanisms include chronic inflammation, psoralen plus ultraviolet therapy (PUVA), and immunomodulatory agents [4]. Although psoriasis is increasingly recognized as a cancer comorbidity, its specific influence on head and neck cancer (HNC) remains underexplored.

HNC ranks as the seventh most common cancer worldwide, particularly in East and Southeast Asia [5, 6]. HNC is a significant health issue in Taiwan, with an incidence of over 22 per 100,000 person-years and a mortality rate of 8.86 per 100,000 person-years, both markedly higher than the global averages of 7.2 and 3.42 per 100,000 person-years, respectively [7, 8]. Despite improvement in diagnosis and treatments, burden of morbidities and mortality of HNC remain significant and the prognosis is worthy of further investigation [9].

Although psoriasis has been linked to certain malignancies, its potential influence on HNC progression and outcomes is unclear. Chronic inflammation and immune dysregulation may promote tumor development, potentially compounded by systemic immunosuppressive therapies that alter the HNC microenvironment. Given the high prevalence of HNC in Taiwan, studying a large patient cohort may offer insights into the potential impact of psoriasis on HNC prognosis and complications. This research seeks to explore these relationships, with the hope of contributing to a better understanding of how psoriasis may affect HNC outcomes.

## Material and methods

### Study population and study design

A retrospective cohort study was conducted at Changhua Christian Hospital (CCH), Taiwan, from January 2010 to August 2021. Eligible patients were those aged 18 years and above and were identified as having HNC based on International Classification of Diseases, Ninth Revision (ICD-9) and Tenth Revision (ICD-10). The ICD codes included oral cancer (C00, C02, C03, C04, C05.0), oropharyngeal cancer (C01, C05.1, C05.2, C09, C10), hypopharyngeal carcinoma (C12, C13), laryngeal cancer(C32), and nasal cavity and sinus cancer (C30, C31). Patients who did not receive HNC treatment or those diagnosed with salivary gland or nasopharyngeal cancer were excluded due to distinct disease courses and prognoses. Eligible subjects were categorized into two cohorts based on psoriasis (ICD-9/ICD-10). In our study, the diagnosis of psoriasis was defined as occurring before the index date, which is the first treatment date for HNC. This ensures that psoriasis was a pre-existing condition before the initiation of HNC treatment. The "psoriasis group" comprised individuals with concurrent diagnoses of both HNC and psoriasis, while the "non-psoriasis group" included patients with HNC but no diagnosis of psoriasis.

Data were retrieved from the Changhua Christian Hospital Clinical Research Database (CCHRD), an integrated repository of electronic medical records, hospitalizations, interventions, prescriptions, laboratory results, clinical visits, and mortality data. Key demographic and clinical parameters included age, sex, Body Mass Index (BMI), smoking, alcohol consumption, betel nut chewing, education level, tumor stage, pathological grade, Eastern Cooperative Oncology Group (ECOG) performance status, and comorbidity profile (hypertension, diabetes mellitus, coronary artery disease, chronic liver disease, chronic kidney disease, chronic lung disease, and previous cancers). Nutritional supplement use (glutamine, amino acids, albumin) was also recorded.

### Study outcomes

The primary endpoint was the 1-year infection event post-HNC treatment which defined as the eligible HNC patients who experience at least one infection event within the first year after initiating HNC treatment. The index date is the first recorded date when an eligible HNC patient received any HNC-related treatment. Infection events are identified using ICD-9-CM codes (001–139) and ICD-10-CM codes (A00–A99, B00–B99) based on electronic medical records, occurring between day 2 and day 365 after the index date. The exclusion of day 1 ensures that infections are assessed post-treatment rather than being pre-existing or immediately associated with treatment initiation. Subgroup comparisons included psoriasis patients on systemic therapy versus those not, and mild versus severe psoriasis. Mild psoriasis was defined as having a body surface area (BSA) ≤ 10% and being treated solely with topical medications, such as topical steroids. Moderate-to-severe psoriasis was classified based on the presence of any of the following criteria: BSA > 10%, psoriasis affecting the face, palms, soles, nails, or genital region, prior use of phototherapy, prescription of systemic therapies, or hospitalization due to psoriasis. Systemic therapies included Acitretin, Methotrexate, Cyclosporine, or biologic agents such as Etanercept, Infliximab, Brodalumab, Ustekinumab, Adalimumab, and Ixekizumab. The secondary outcomes were overall survival rate and disease-free survival rate, which were compared between the psoriasis and non-psoriasis cohorts among HNC patients.

### Statistical analysis

Data were expressed as numbers (proportions) for categorical variables and means ± standard deviations (SD) for continuous variables. Categorical variables were compared using chi-square tests, and continuous variables with Student’s t-tests. Crude and multivariate Cox proportional hazard models with Firth’s penalized likelihood were employed to assess infection risk between psoriasis and non-psoriasis groups, as well as the impact of systemic therapy and psoriasis severity. A penalized likelihood approach was used to reduce parameter estimation bias caused by the small sample size. Kaplan–Meier analysis examined 1-year cumulative infection rates, long-term survival, and disease-free survival, with log-rank tests evaluating group differences.

Propensity Score Matching (PSM) in a 1:10 ratio used a non-parsimonious multivariate logistic regression and nearest-neighbor matching (caliper = 0.1 SD). To enhance the robustness of our study findings, a sensitivity analysis was implemented to scrutinize the relationship between the definition of infection as per ICD diagnostic codes and the use of antibacterial drugs, classified under ATC code J01. The statistically significant was defined as a *p*-value < 0.05. This study was approved by the Institutional Review Board (IRB) of CCH (approval number 230203).

## Results

**Fig. 1:**
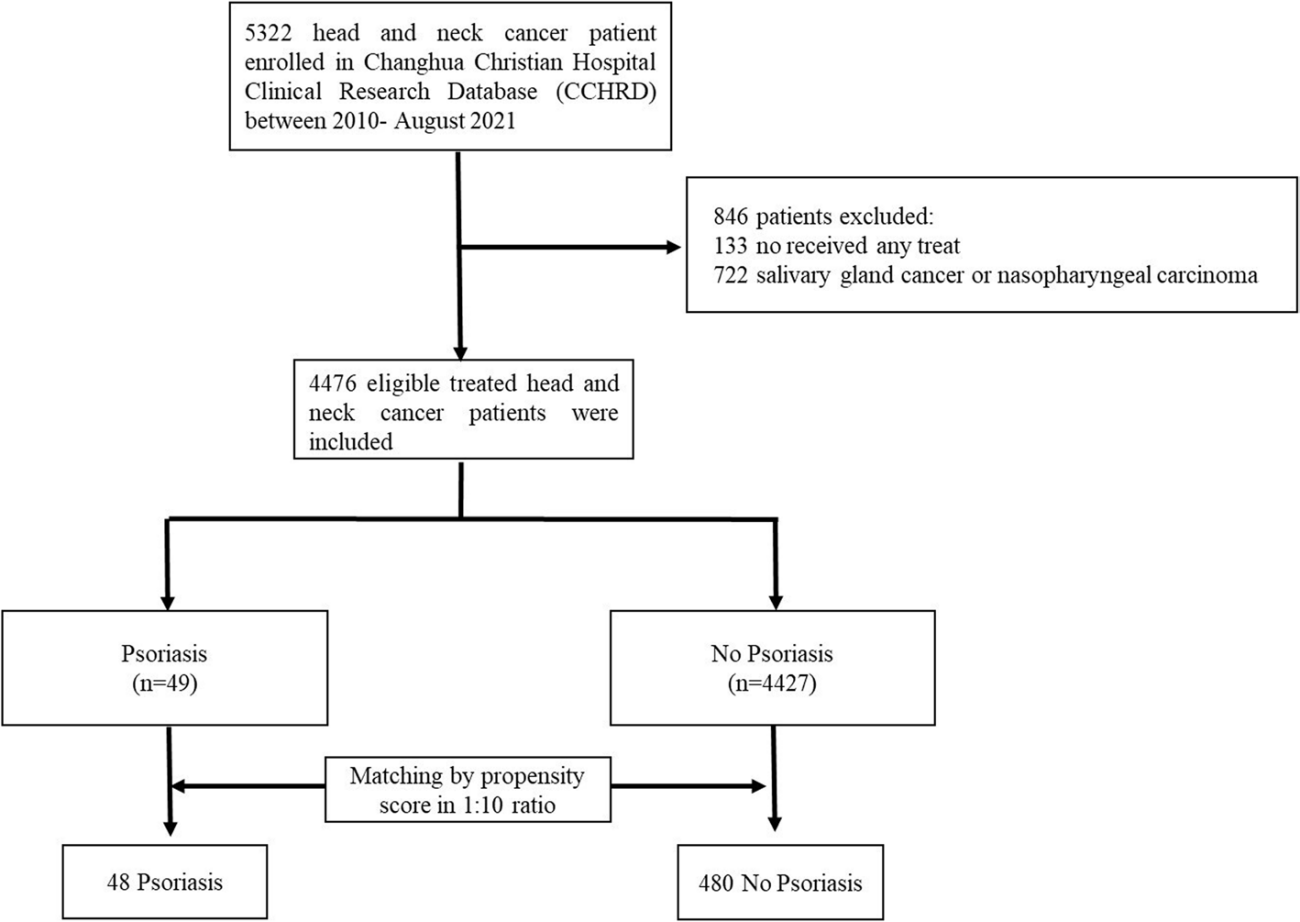
Flowchart of study population selection process. A total of 5322 patients with head and neck cancer (HNC) were enrolled from the Changhua Christian Hospital Clinical Research Database (CCHRD) between January 2010 and August 2021. After excluding 846 patients (133 who did not receive treatment and 722 with salivary gland cancer or nasopharyngeal carcinoma), 4476 eligible patients were included in the final analysis. These patients were divided into two cohorts: 49 patients in the psoriasis group and 4427 patients in the non-psoriasis group. Matching by propensity score in a 1:10 ratio resulted in 48 patients in the psoriasis group and 480 patients in the non-psoriasis group

A total of 5,322 patients diagnosed with HNC between January 2010 and August 2021 were initially included. After applying exclusion criteria, 133 patients who did not receive treatment and 722 patients with salivary gland or nasopharyngeal cancer were excluded. The remaining 4,476 eligible patients were categorized into two groups: the psoriasis group (n = 49) and the non-psoriasis group (n = 4,427). PSM was performed in a 1:10 ratio to balance the groups. After matching, the psoriasis group included 48 patients, and the non-psoriasis group included 480 patients. Figure 1 provides an overview of the patient selection process.

Table 1 presents the baseline characteristics of the patients. The majority of patients in both groups were male, with 4,185 (94.5%) in the non-psoriasis group and 48 (98%) in the psoriasis group. No significant differences were found between the two groups in terms of sex, age, BMI, smoking habits, alcohol consumption, betel nut chewing, education level, ECOG score, tumor size, cancer stage, pathological grading, presence of comorbidities, and nutrition supplement usage (*P* > 0.05). The cumulative infection rate within one year after treatment was 996 out of 4,427 patients (22.5%) in the non-psoriasis group and 16 out of 49 patients (32.7%) in the psoriasis group, with a *p*-value of 0.091, indicating no statistically significant difference before matching. After performing PSM, a significant difference emerged between the groups: the psoriasis group had 16 cases (33.3%), while the non-psoriasis group had 97 cases (20.2%), with a *p*-value of 0.035 (Table 1).

**Table 1:** Baseline characteristics of head and neck cancer patients with and without psoriasis, before and after propensity score matching

Characteristic	Before propensity score matching			After propensity score matching		
	No Psoriasis	Psoriasis	*P*-value	No Psoriasis	Psoriasis	*P*-value
	(*N* = 4427)	(*N* = 49)		(*N* = 480)	(*N* = 48)	
Gender, Male, *N* (%)	4185 (94.5)	48 (98)	0.293	472 (98.3)	47 (97.9)	0.579
Age (Mean ± SD)	58.5 ± 11.4	60.9 ± 9	0.072	60.9 ± 11.1	61 ± 9.1	0.941
BMI (Mean ± SD)	23.8 ± 4.1	24.1 ± 4.2	0.637	23.9 ± 4	24.2 ± 4.2	0.658
Smoking habits						
None, *N* (%)	334 (7.5)	1 (2)	0.346	17 (3.5)	1 (2.1)	0.816
Quit, *N* (%)	3256 (73.5)	38 (77.6)		354 (73.8)	37 (77.1)	
Current, *N* (%)	837 (18.9)	10 (20.4)		109 (22.7)	10 (20.8)	
Betel nut habits						
None, *N* (%)	324 (7.3)	1 (2)	0.236	14 (2.9)	1 (2.1)	0.713
Quit, *N* (%)	2654 (60)	28 (57.1)		251 (52.3)	28 (58.3)	
Current, *N* (%)	1449 (32.7)	20 (40.8)		215 (44.8)	19 (39.6)	
Alcohol habits						
None, *N* (%)	3683 (83.2)	38 (77.6)	0.294	383 (79.8)	37 (77.1)	0.657
Current, *N* (%)	744 (16.8)	11 (22.4)		97 (20.2)	11 (22.9)	
ECOG						
0, *N* (%)	3883 (87.7)	44 (89.8)	0.681	438 (91.3)	43 (89.6)	0.699
> = 1, *N* (%)	544 (12.3)	5 (10.2)		42 (8.8)	5 (10.4)	
Tumor size, mm (Mean ± SD)	29.1 ± 18.2	25.4 ± 18.2	0.157	25.7 ± 15.9	26 ± 17.9	0.905
Stage						
I, *N* (%)	1336 (30.2)	18 (36.7)	0.714	161 (33.5)	17 (35.4)	0.980
II, *N* (%)	662 (15)	7 (14.3)		81 (16.9)	7 (14.6)	
III, *N* (%)	482 (10.9)	6 (12.2)		59 (12.3)	6 (12.5)	
IV, *N* (%)	1947 (44)	18 (36.7)		179 (37.3)	18 (37.5)	
Lymphatic invasion (%)	1183 (26.7)	10 (20.4)	0.320	126 (26.3)	10 (20.8)	0.491
Comorbidity^a^						
None, *N* (%)	2153 (48.6)	17 (34.7)	0.160	181 (37.7)	17 (35.4)	0.966
One, *N* (%)	1338 (30.2)	17 (34.7)		147 (30.6)	17 (35.4)	
Two, *N* (%)	649 (14.7)	9 (18.4)		105 (21.9)	9 (18.8)	
More than three, *N* (%)	287 (6.5)	6 (12.2)		47 (9.8)	5 (10.4)	
postoperative R/T	1395(31.5%)	11(22.4%)	0.228	137(28.5%)	11(22.9%)	0.408
postoperative CCRT	780(17.6%)	7(14.3%)	0.674	64(13.3%)	7(14.6%)	0.809
**Tumor site**			0.866			0.949
Oral cavity	2854(64.5%)	31(63.3%)		214(79.3%)	21(77.8%)	
Oropharyngeal	657(14.8%)	9(18.4%)		33(12.2%)	3(11.1%)	
Hypopharyngeal	518(11.7%)	6(12.2%)		10(3.7%)	1(3.7%)	
Laryngeal cancer	333(7.5%)	3(6.1%)		13(4.8%)	2(7.4%)	
Nasal cavity and sinus	65(1.5%)	0(0%)		0(0%)	0(0%)	
Propensity score (Mean ± SD)	0.011 ± 0.008	0.016 ± 0.008	< 0.001*	0.015 ± 0.007	0.015 ± 0.007	0.997
Infection within 1 year after treatment, *N* (%)	996 (22.5)	16 (32.7)	0.091	97 (20.2)	16 (33.3)	0.035*

Abbreviation: *SD *standard deviation, *ECOG *Eastern Cooperative Oncology Group, *PSM *propensity score matching, *BMI *body mass index

^a^Comorbidities were categorized based on the number of diseases, including hypertension, diabetes mellitus, coronary artery disease, chronic liver disease, chronic kidney disease, chronic lung disease, and cancer

^*^ *P*-values reflect statistical significance, with significance defined as *P* < 0.05

Table 2 presents the results of the unadjusted and adjusted Cox proportional hazards regression model, along with the PSM analysis for infection risk in psoriasis patients with HNC. The crude hazard ratio (HR) for the psoriasis group compared to the non-psoriasis group was 1.65 (95% CI 1.01–2.68; *p* = 0.044), indicating a significantly higher risk of infection in the psoriasis group before adjustments. After adjusting for confounding factors, the adjusted HR was 1.66 (95% CI: 1.02–2.71; *p* = 0.041), confirming the increased infection risk in the psoriasis group. Following PSM, the HR further increased to 1.84 (95% CI: 1.09–3.11; *p* = 0.023), demonstrating a statistically significant elevation in infection risk for patients with psoriasis compared to those without.

**Table 2:** Unadjusted and Adjusted Cox Proportional Regression Model and PSM Results for Infection Risk in Psoriasis Patients with HNC

	Crude HR (95% CI)	*P*-value	aHR (95% CI)	*P*-value	PSM (95% CI)	*P*-value
**Psoriasis**						
No	1 (reference)		1 (reference)		1 (reference)	
Yes	1.65 (1.01,2.68)	0.044*	1.66 (1.02,2.71)	0.041*	1.84 (1.09,3.11)	0.023*
**Psoriasis therapy**						
No Psoriasis	1 (reference)		1 (reference)		1 (reference)	
Psoriasis without systemic drug therapy	1.50 (0.53,4.29)	0.448	1.49 (0.52,4.27)	0.455	1.62 (0.55,4.73)	0.382
Psoriasis with systemic drug therapy	1.76 (1.03,3.01)	0.040*	1.79 (1.00,30.21)	0.050	1.99 (1.12,3.53)	0.019*
*** P for trend***		0.091		0.113		0.049*
**Severity of psoriasis**						
No Psoriasis	1 (reference)		1 (reference)		1 (reference)	
Mild psoriasis	1.23 (0.53,2.85)	0.627	1.49 (0.65,3.46)	0.349	1.40 (0.59,3.32)	0.448
Severe psoriasis	2.08 (1.16,3.72)	0.014*	1.87 (0.98,3.55)	0.056	2.29 (1.24,4.24)	0.009*
*** P for trend***		0.043*		0.105		0.027*

Abbreviation: *HR *Hazard ratio, *aHR *adjusted hazard ratio, *PSM *propensity score match, *95% CI *95% confidence interval

^*^ *P*-values reflect statistical significance, with significance defined as *P* < 0.05

Additionally, a subgroup analysis was conducted within the psoriasis group based on the use of systemic therapy and the severity of psoriasis. Prior to PSM, the subgroup receiving systemic therapy demonstrated a significantly higher infection risk compared to the non-psoriasis group, with a crude HR of 1.76 (95% CI: 1.03–3.01; *P* = 0.040, p for trend = 0.091). After PSM, the psoriasis group receiving systemic therapy continued to show a significantly increased risk of infection within one year, with a hazard ratio of 1.99 (95% CI: 1.12–3.53; *p* = 0.019, p for trend = 0.049). Based on the severity of psoriasis, the mild psoriasis group did not show a significant increase in hazard ratio compared to the non-psoriasis group, while the severe psoriasis group exhibited a significantly higher infection rate within one year. The crude hazard ratio for severe psoriasis was 2.08 (95% CI: 1.16–3.72; *P* = 0.014), which increased to 2.29 (95% CI: 1.24–4.24; *p* = 0.009) after propensity score matching, as shown in Table 2.

**Fig. 2:**
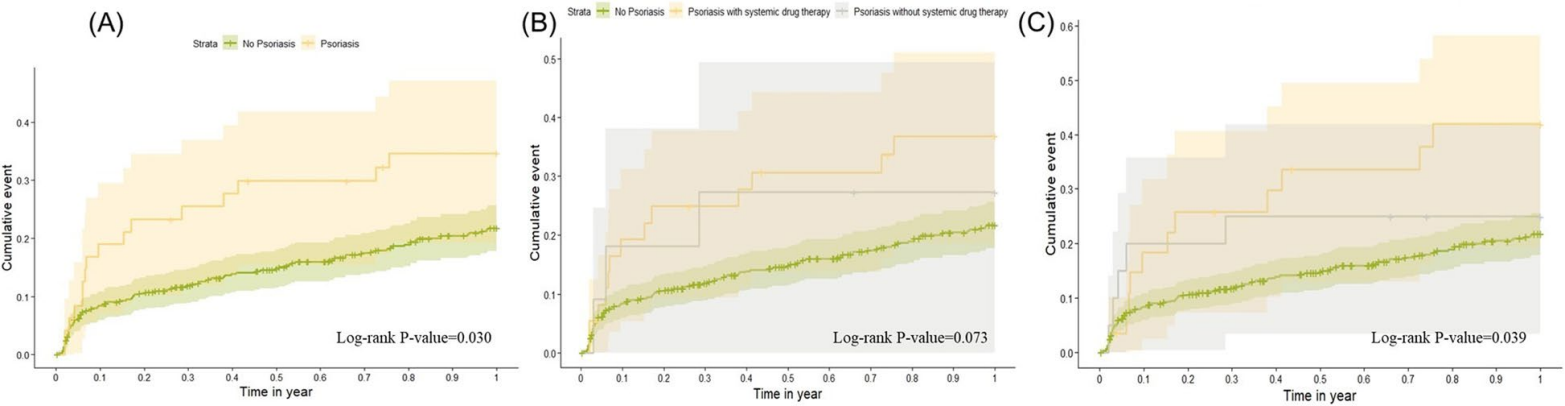
Cumulative Incidence Rate of Infection Within One Year After Treatment for HNC Patients Stratified by Psoriasis Status. **A** Comparison of cumulative infection events between the psoriasis group and the non-psoriasis group (Log-rank *P*-value = 0.030). **B** Comparison between HNC patients with psoriasis receiving systemic drug therapy, those without systemic drug therapy, and non-psoriasis patients (Log-rank *P*-value = 0.073). **C** Comparison of cumulative infection events between severe psoriasis, mild psoriasis, and non-psoriasis groups (Log-rank *P*-value = 0.039)

Figure 2 illustrates the Kaplan–Meier analysis of cumulative infection rates within one year. Figure 2A demonstrates that patients in the psoriasis group had a significantly higher cumulative infection rate compared to the non-psoriasis group (Log-rank *P* = 0.030). Figure 2B compares the cumulative infection rates among psoriasis patients, stratified by the use of systemic treatments, and the non-psoriasis group, showing no statistically significant difference in infection rates among the three groups (Log-rank *P* = 0.073). Figure 2C stratifies psoriasis patients by disease severity, revealing a significant difference in infection rates among the three psoriasis status groups (Log-rank *P* = 0.039).

**Fig. 3:**
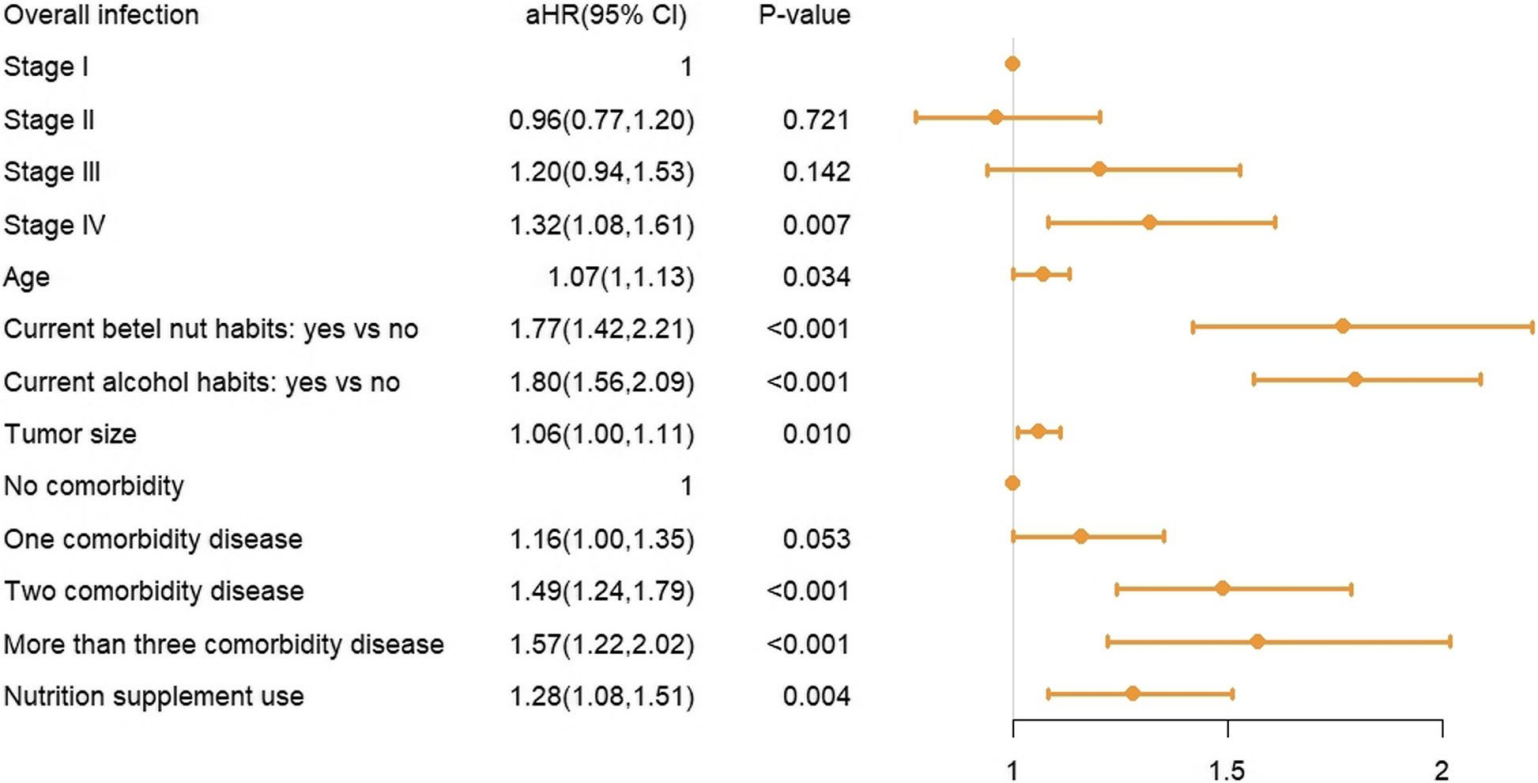
Forest Plot for Significant Risk Factors of Infection in Head and Neck Cancer Patients. The forest plot illustrates the aHR and 95% CI for various risk factors associated with infection in HNC patients. The risk factors include cancer stage, age, betel nut and alcohol consumption, tumor size, comorbidity status, and the use of nutritional supplements. Significant risk factors for increased infection risk include stage IV cancer (aHR: 1.32, 95% CI: 1.08–1.61, *P* = 0.007), older age (aHR: 1.07, 95% CI: 1.01–1.13, *P* = 0.034), current betel nut habits (aHR: 1.77, 95% CI: 1.42–2.21, *P* < 0.001), current alcohol consumption (aHR: 1.80, 95% CI: 1.56–2.09, *P* < 0.001), larger tumor size (aHR: 1.06, 95% CI: 1.00–1.11, *P* = 0.010), having two comorbidities (aHR: 1.49, 95% CI: 1.24–1.79, *P* < 0.001), and more than three comorbidities (aHR: 1.57, 95% CI: 1.22–2.02, *P* < 0.001)

In the analysis of risk factors, several factors were found to be significantly associated with a higher infection rate. These included current betel nut chewing (adjusted hazard ratio [aHR] = 1.77, 95% CI: 1.42–2.21, *P* < 0.001), current alcohol consumption (aHR = 1.80, 95% CI: 1.56–2.09, *P* < 0.001), having two comorbidities (aHR = 1.49, 95% CI: 1.24–1.79, *P* < 0.001), and having more than two comorbidities (aHR = 1.57, 95% CI: 1.22–2.02, *P* < 0.001). Tumor size (aHR = 1.06, 95% CI: 1.00–1.11, *P* = 0.010) and nutrition supplement use (aHR = 1.28, 95% CI: 1.08–1.51, *P* = 0.004) were also associated with an increased infection risk. The forest plot illustrating these associations is presented in Fig. 3.

**Fig. 4:**
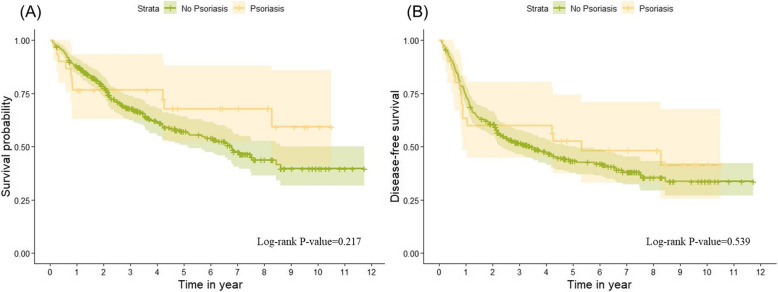
Overall Survival and Disease-Free Survival Curves for Stage I to III Head and Neck Cancer Patients Stratified by Psoriasis Status in a Propensity Score Matched Cohort. (A) Overall survival curve comparing patients with and without psoriasis, showing no statistically significant difference (Log-rank *P* = 0.217). (B) Disease-free survival curve, indicating no significant difference between the psoriasis and non-psoriasis groups (Log-rank *P* = 0.539). Both analyses were conducted on stage I to III HNC patients using propensity score matching

In the analysis of the 10-year survival curves between patients with psoriasis and those without psoriasis in the HNC cohort, there was no significant difference observed (Log-rank *P* = 0.217). Additionally, psoriasis did not significantly affect the disease-free survival of patients with stage I to III head and neck cancer (Log-rank *P* = 0.539). These findings are illustrated in Fig. 4, where the Kaplan–Meier plots for 10-year survival and disease-free survival are presented, respectively.

In Table 3, the sensitivity analysis, which incorporates antibacterial drug use, confirms a higher infection risk in psoriasis patients compared to non-psoriasis patients. The proportion of antibacterial drug use was higher in the psoriasis group (93.75%) than in the non-psoriasis group (84.94%). Despite this, the psoriasis group exhibited a significantly elevated infection risk, with a crude HR of 1.81 (95% CI: 1.09–2.99; *P* = 0.021). This risk remained significant after adjustment (aHR = 1.78, 95% CI: 1.02–3.08; *P* = 0.041) and increased further after propensity score matching (HR = 2.04, 95% CI: 1.18–3.51; *P* = 0.011).

**Table 3:** Sensitivity Analysis of Infection^a^ Risk Based on Antibacterial Drug Use with ATC Code and ICD Diagnosis Codes

Characteristic	Antibacterial Drug Use (n)	Total Patients (N)	Proportion (%)	Crude HR (95% CI)	*P*-value	aHR^1^ (95% CI)	*P*-value	PSM (95% CI)	*P*-value
**Psoriasis**	**861**	**1012**	**85.08**						
No	846	996	84.94	1 (reference)		1 (reference)		1 (reference)	
Yes	15	16	93.75	1.81(1.09,2.99)	0.021*	1.78(1.02,3.08)	0.041*	2.04(1.18,3.51)	0.011*

Abbreviation: *HR* Hazard ratio, *CI* Confidence interval, *aHR* Adjusted hazard ratio in model, *PSM *Propensity score matching, *ATC* Anatomical Therapeutic Chemical Classification System

^a^The definition of infection was based on both ICD diagnosis codes and the use of antibacterial drugs with ATC code J01

^*^P-values reflect statistical significance, with significance defined as *P* < 0.05

## Discussion

This study provides novel insights into the impact of psoriasis on infection risk and long-term outcomes in HNC patients. Our findings indicate a significantly higher infection risk within one year post-treatment for HNC in patients with psoriasis compared to those without, particularly in those receiving systemic therapy. However, psoriasis did not have a significant effect on long-term survival outcomes, as shown in both overall and disease-free survival analyses. The sensitivity analysis further reinforced the robustness of these findings, demonstrating that the increased infection risk persisted even after accounting for antibacterial drug use. To our knowledge, this is the first study to specifically examine the influence of psoriasis on HNC prognosis, providing important evidence for clinicians managing patients with coexisting psoriasis and HNC. These results suggest the need for heightened infection surveillance in this population, particularly during the first year after treatment.

Previous study by Huh et al. have demonstrated that psoriasis increases the overall risk of head and neck cancers, with an overall hazard ratio of 1.72 for all head and neck cancer sites combined [10]. However, the investigation primarily focusses on cancer incidence. Our study builds on this by exploring how psoriasis influences post-treatment infection risk and survival. While several prognostic factors, such as tumor site, stage, smoking history, and nutritional status, have been studied previously [11, 12], the impact of psoriasis on head and neck cancer survival remains largely unexplored. In the broader context of psoriasis and cancer mortality, the meta-analysis from JAMA Dermatology found no significant increase in overall cancer mortality risk for people with psoriasis, with a pooled relative risk (RR) of 1.05 (95% CI, 0.96–1.16). However, for severe psoriasis, the cancer mortality risk significantly increases, with a pooled RR of 1.22 (95% CI, 1.08–1.38). Specific cancers, such as esophageal (RR 2.53), liver (RR 1.43), and pancreatic cancers (RR 1.31), showed particularly elevated risks, although this analysis did not include head and neck cancers [3]. Several nationwide population-based studies have demonstrated an increased cancer mortality risk in psoriasis patients, particularly those with severe cases [13–16]. In the Swedish study by Svedbom et al., severe psoriasis patients showed a cancer mortality hazard ratio (HR) above 1.0, indicating elevated risk compared to controls, while mild psoriasis patients did not face significantly higher risks. Similarly, in Taiwan, Lee et al. found the highest cause-specific mortality in malignancies for severe psoriasis (SMR 1.53) [13, 15]. Salahadeen et al. also noted increased cancer mortality in severe psoriasis cases [14]. Our study on head and neck cancer yielded similar results for overall survival, showing no significant difference between psoriasis and non-psoriasis patients. However, due to limited case numbers, we could not stratify by psoriasis severity, limiting further analysis. Future research should focus on whether severe psoriasis has a similar impact on head and neck cancer prognosis as seen in other cancer types.

Our study revealed that patients with psoriasis had a higher risk of infection following treatment for head and neck cancer compared to those without psoriasis. This trend was particularly evident in patients with severe psoriasis and those receiving systemic therapy. Based on this finding, we aimed to further explore the reasons behind the higher cumulative infection rates in patients with psoriasis, considering whether this increased risk stems from the disease itself or from the use of immunosuppressive agents commonly prescribed to psoriasis patients.

Psoriasis is an immune-mediated chronic inflammatory disease characterized by abnormal keratinocyte proliferation and the involvement of multiple immune cells such as T cells, dendritic cells, and neutrophils [17]. Additionally, various cytokines have been implicated in the pathogenesis of psoriasis, involving in the modulation of immunity via interaction with other cytokines, chemokines, and immune cells [18, 19]. This immune dysregulation results in elevated inflammatory responses, contributing to both psoriasis pathogenesis and increased susceptibility to infections [20]. Various cytokines and immune pathways implicated in psoriasis have led to the use of systemic immunosuppressive therapies, which may further heighten infection risks in treated patients [21]. Multiple studies have demonstrated an increased risk of serious infections in psoriasis patients compared to those without the disease. A UK-based study reported a 36% higher risk of severe infections in psoriasis patients, with a correlation between disease severity and infection risk [22]. Similarly, a Taiwanese study found that psoriasis patients had a significantly higher risk of severe infections (aHR: 1.21), with even greater risks for those with moderate to severe psoriasis (aHR: 1.30). Additionally, infection-related mortality was also elevated (aHR: 1.15), particularly in moderate to severe cases (aHR: 1.39) [23]. Both studies identified elevated risks for respiratory, skin, soft-tissue, and urinary tract infections. A US-based study specifically examining biologic therapies found that psoriasis patients using biologics had a 31% higher risk of serious infection compared to those using non-biologic systemic treatments, with particularly increased risks for skin or soft-tissue infection [24]. The mechanism underlying the increased infection rate due to psoriasis remains unclear. However, it is known that psoriasis leads to disrupted skin barrier, particularly due to epidermal physical barrier dysregulation resulting from abnormal keratinocyte proliferation [25]. Woo et al. (2020) emphasized that psoriasis patients face increased infection risks due to several underlying mechanisms. Immune dysregulation, particularly the elevated levels of cytokines like TNF-α and IL-6, impairs the body's pathogen response, while the disrupted skin barrier in psoriatic lesions facilitates pathogen entry. Comorbidities like diabetes and cardiovascular disease, along with lifestyle factors such as smoking and alcohol consumption, further weaken the immune system. Additionally, immunosuppressive therapies targeting TNF-α, IL-17, and IL-23, and genetic predispositions also heighten the risk of infections [26–29]. Take Cyclosporin(CsA) for example, in addition to being a common systemic therapy for psoriasis, is also widely used in the prevention and treatment of organ transplant rejection and also in autoimmune diseases. CsA inhibits dendritic cell production of IL-2. Upon stimulation by pathogen-associated molecular patterns (PAMPs), dendritic cells rapidly produce IL-2 to stimulate T cell proliferation. Therefore, inhibiting this process disrupts subsequent adaptive immune responses. Furthermore, CsA also reduces the secretion of pro-inflammatory cytokines such as TNF-α. These effects collectively diminish the protective ability against pathogens, thus increasing the risk of infection [30]. Biologics such as anti-IL-17, anti-IL-23 are also an important treatment option for psoriasis. However, they also play a crucial immune role in defending against external pathogens. Many immune cells respond to IL-23, including adaptive Th17 cells, natural Th17 cells, and innate lymphoid cells, which accumulate in non-lymphoid tissues. When the tissue is infected or injured, this leads to a localized inflammatory response. The above process also heavily relies on IL-17-mediated mechanisms [31]. In the study assessing 7335 patients by Deodhar A et al., the incidence rate of serious infections in PsO and PsA patients using Secukinumab was 1.4 and 1.9 per 100 patient-years, respectively [32]. Methotrexate exerts mild-to-moderate immunosuppressive effects by inhibiting T- and B-lymphocyte proliferation, which can lead to increased susceptibility to common bacterial and viral infections, as well as occasional opportunistic pathogens such as Pneumocystis jirovecii [33, 34].

This study has some strengths, including its large cohort size and comprehensive analysis, which allowed for robust examination of psoriasis' impact on infection risk in HNC patients. The use of sensitivity analysis further ensured the reliability of our findings. However, this study has several limitations that warrant consideration. The retrospective nature of the design introduces potential selection bias and limits our ability to establish causality. To address this, we implemented rigorous statistical adjustments, such as PSM, to minimize confounding factors. Another limitation is the reliance on ICD codes for infection identification, which may introduce misclassification bias. We attempted to mitigate this by performing a sensitivity analysis, incorporating antibacterial drug use to improve accuracy. Additionally, the findings may have limited generalizability as they are based on a Taiwanese population, which, despite having one of the highest global prevalence rates of HNC, offering a degree of representativeness. Despite being a single-center study, Changhua Christian Hospital accounts for approximately 8% of all head and neck cancer cases in Taiwan, provides meaningful insights. However, the results should still be interpreted with caution, and further multi-center research is needed to confirm their generalizability. Lastly, potential unmeasured confounders, such as genetic predispositions, may have influenced the results. While we included clinical and demographic variables, some factors remain beyond the scope of this study. Future research should address these gaps to strengthen the understanding of infection risks in psoriasis patients.

## Conclusion

This study highlights a significantly increased risk of infection in HNC patients with psoriasis, particularly those receiving systemic therapy, while showing no substantial impact on long-term survival outcomes. These findings underscore the importance of tailored clinical management for HNC patients with concurrent psoriasis, with heightened infection surveillance and precautionary measures. Future research is needed to further explore the mechanisms driving these outcomes and to optimize treatment strategies for this high-risk population.

## Data Availability

The data that support the findings of this study are available from the corresponding author upon reasonable request.

## References

[1] Blauvelt A, Chiricozzi A. The immunologic role of IL-17 in psoriasis and psoriatic arthritis pathogenesis. Clin Rev Allergy Immunol. 2018;55(3):379–90. 30109481 10.1007/s12016-018-8702-3 PMC6244934

[2] Tang ZL, Zhang K, Lv SC, Xu GW, Zhang JF, Jia HY. LncRNA MEG3 suppresses PI3K/AKT/mTOR signalling pathway to enhance autophagy and inhibit inflammation in TNF-α-treated keratinocytes and psoriatic mice. Cytokine. 2021;148: 155657. 34425525 10.1016/j.cyto.2021.155657

[3] Trafford AM, Parisi R, Kontopantelis E, Griffiths CEM, Ashcroft DM. Association of psoriasis with the risk of developing or dying of cancer: a systematic review and meta-analysis. JAMA Dermatol. 2019;155(12):1390–403. 31617868 10.1001/jamadermatol.2019.3056 PMC6802036

[4] Rademaker M, Rubel DM, Agnew K, Andrews M, Armour KS, Baker C, et al. Psoriasis and cancer. an Australian/New Zealand narrative. Australas J Dermatol. 2019;60(1):12–8. 29992535 10.1111/ajd.12889

[5] Rettig EM, D’Souza G. Epidemiology of head and neck cancer. Surg Oncol Clin N Am. 2015;24(3):379–96. 25979389 10.1016/j.soc.2015.03.001

[6] Sung H, Ferlay J, Siegel RL, Laversanne M, Soerjomataram I, Jemal A, et al. Global cancer statistics 2020: GLOBOCAN estimates of incidence and mortality worldwide for 36 cancers in 185 countries. CA Cancer J Clin. 2021;71(3):209–49. 33538338 10.3322/caac.21660

[7] Padua PF, Lin CC, Chien HT, Young CK, Kuo CF, See LC, et al. Familial aggregation of head and neck cancer in Taiwan. Laryngoscope. 2021;131(4):806–12. 32820835 10.1002/lary.28992

[8] Siegel RL, Miller KD, Fuchs HE, Jemal A. Cancer Statistics, 2021. CA Cancer J Clin. 2021;71(1):7–33. 33433946 10.3322/caac.21654

[9] Sasahira T, Kirita T. Hallmarks of cancer-related newly prognostic factors of oral squamous cell carcinoma. Int J Mol Sci. 2018;19(8):2413. 30115834 10.3390/ijms19082413 PMC6121568

[10] Huh G, Kim D, Lee KN, Han K, Cho JH. Risk of head and neck cancer in patients with psoriasis: a nationwide population-based study. Acta Derm Venereol. 2024;104:adv18487. 38757177 10.2340/actadv.v104.18487 PMC11131588

[11] Du E, Mazul AL, Farquhar D, Brennan P, Anantharaman D, Abedi-Ardekani B, et al. Long-term survival in head and neck cancer: impact of site, stage, smoking, and human papillomavirus status. Laryngoscope. 2019;129(11):2506–13. 30637762 10.1002/lary.27807 PMC6907689

[12] Wang EY, Chen MK, Hsieh MY, Kor CT, Liu YT. Relationship between preoperative nutritional status and clinical outcomes in patients with head and neck cancer. Nutrients. 2022;14(24):5331. 36558490 10.3390/nu14245331 PMC9782741

[13] Lee MS, Yeh YC, Chang YT, Lai MS. All-cause and cause-specific mortality in patients with psoriasis in taiwan: a nationwide population-based study. J Invest Dermatol. 2017;137(7):1468–73. 28257796 10.1016/j.jid.2017.01.036

[14] Salahadeen E, Torp-Pedersen C, Gislason G, Hansen PR, Ahlehoff O. Nationwide population-based study of cause-specific death rates in patients with psoriasis. J Eur Acad Dermatol Venereol. 2015;29(5):1002–5. 24909271 10.1111/jdv.12523

[15] Svedbom A, Dalén J, Mamolo C, Cappelleri JC, Mallbris L, Petersson IF, et al. Increased cause-specific mortality in patients with mild and severe psoriasis: a population-based Swedish register study. Acta Derm Venereol. 2015;95(7):809–15. 25766866 10.2340/00015555-2095

[16] Abuabara K, Azfar RS, Shin DB, Neimann AL, Troxel AB, Gelfand JM. Cause-specific mortality in patients with severe psoriasis: a population-based cohort study in the U.K. British Journal of Dermatology. 2010;163(3):586–92. 20633008 10.1111/j.1365-2133.2010.09941.x PMC2966545

[17] Armstrong AW, Read C. Pathophysiology, clinical presentation, and treatment of psoriasis: a review. JAMA. 2020;323(19):1945–60. 32427307 10.1001/jama.2020.4006

[18] Grän F, Kerstan A, Serfling E, Goebeler M, Muhammad K. Current Developments in the Immunology of Psoriasis. Yale J Biol Med. 2020;93(1):97–110. 32226340 PMC7087066

[19] Shimoura N, Nagai H, Fujiwara S, Jimbo H, Yoshimoto T, Nishigori C. Interleukin (IL)-18, cooperatively with IL-23, induces prominent inflammation and enhances psoriasis-like epidermal hyperplasia. Arch Dermatol Res. 2017;309(4):315–21. 28299442 10.1007/s00403-017-1735-2

[20] Lanna C, Mancini M, Gaziano R, Cannizzaro MV, Galluzzo M, Talamonti M, et al. Skin immunity and its dysregulation in psoriasis. Cell Cycle. 2019;18(20):2581–9. 31416396 10.1080/15384101.2019.1653099 PMC6773242

[21] Higashi Y, Imafuku S, Tsuruta N, Murotani K. Systemic therapy for psoriasis and the risk of cutaneous infections. J Dermatol. 2024;51(7):939–49. 38660962 10.1111/1346-8138.17245 PMC11483952

[22] Yiu ZZN, Parisi R, Lunt M, Warren RB, Griffiths CEM, Langan SM, et al. Risk of hospitalization and death due to infection in people with psoriasis: a population-based cohort study using the Clinical Practice Research Datalink. Br J Dermatol. 2021;184(1):78–86. 32222069 10.1111/bjd.19052

[23] Chen TC, Wang TC, Yiu ZZN, Lee MS, Chen LC, Chan KA, et al. Risk of serious infection and infection mortality in patients with psoriasis: a nationwide cohort study using the Taiwan National Health Insurance claims database. J Eur Acad Dermatol Venereol. 2024;38(1):136–44. 37611288 10.1111/jdv.19466

[24] Dobry AS, Quesenberry CP, Ray GT, Geier JL, Asgari MM. Serious infections among a large cohort of subjects with systemically treated psoriasis. J Am Acad Dermatol. 2017;77(5):838–44. 28917384 10.1016/j.jaad.2017.07.047 PMC5880275

[25] Orsmond A, Bereza-Malcolm L, Lynch T, March L, Xue M. Skin barrier dysregulation in psoriasis. Int J Mol Sci. 2021;22(19):10841. 34639182 10.3390/ijms221910841 PMC8509518

[26] Woo YR, Park CJ, Kang H, Kim JE. The risk of systemic diseases in those with psoriasis and psoriatic arthritis: from mechanisms to clinic. Int J Mol Sci. 2020;21(19):7041. 32987907 10.3390/ijms21197041 PMC7583918

[27] Li X, Andersen KM, Chang HY, Curtis JR, Alexander GC. Comparative risk of serious infections among real-world users of biologics for psoriasis or psoriatic arthritis. Ann Rheum Dis. 2020;79(2):285–91. 31672774 10.1136/annrheumdis-2019-216102 PMC6989349

[28] Yiu ZZN, Smith CH, Ashcroft DM, Lunt M, Walton S, Murphy R, et al. Risk of Serious Infection in Patients with Psoriasis Receiving Biologic Therapies: A Prospective Cohort Study from the British Association of Dermatologists Biologic Interventions Register (BADBIR). J Invest Dermatol. 2018;138(3):534–41. 29054603 10.1016/j.jid.2017.10.005 PMC5832757

[29] Fowler E, Ghamrawi RI, Ghiam N, Liao W, Wu JJ. Risk of tuberculosis reactivation during interleukin-17 inhibitor therapy for psoriasis: a systematic review. J Eur Acad Dermatol Venereol. 2020;34(7):1449–56. 32012384 10.1111/jdv.16254

[30] Liddicoat AM, Lavelle EC. Modulation of innate immunity by cyclosporine A. Biochem Pharmacol. 2019;163:472–80. 30880061 10.1016/j.bcp.2019.03.022

[31] Gaffen SL, Jain R, Garg AV, Cua DJ. The IL-23-IL-17 immune axis: from mechanisms to therapeutic testing. Nat Rev Immunol. 2014;14(9):585–600. 25145755 10.1038/nri3707 PMC4281037

[32] Deodhar A, Mease PJ, McInnes IB, Baraliakos X, Reich K, Blauvelt A, Leonardi C, Porter B, Das Gupta A, Widmer A, Pricop L, Fox T. Long-term safety of secukinumab in patients with moderate-to-severe plaque psoriasis, psoriatic arthritis, and ankylosing spondylitis: integrated pooled clinical trial and post-marketing surveillance data. Arthritis Res Ther. 2019;21(1):111. 31046809 10.1186/s13075-019-1882-2 PMC6498580

[33] Conway R, Low C, Coughlan RJ, O’Donnell MJ, Carey JJ. Methotrexate and lung disease in rheumatoid arthritis: a meta-analysis of randomized controlled trials. Arthritis and rheumatology (Hoboken, NJ). 2014;66(4):803–12. 24757133 10.1002/art.38322

[34] Kaneko Y, Suwa A, Ikeda Y, Hirakata M. Pneumocystis jiroveci pneumonia associated with low-dose methotrexate treatment for rheumatoid arthritis: report of two cases and review of the literature. Mod Rheumatol. 2006;16(1):36–8. 16622722 10.1007/s10165-005-0443-5

